# Editorial: The isotypes of *α*, *β* and *γ* tubulin: From evolutionary origins to roles in metazoan development and ligand binding differences

**DOI:** 10.3389/fcell.2023.1176739

**Published:** 2023-03-28

**Authors:** Jeffrey Moore, Richard Luduena, Jack A. Tuszynski

**Affiliations:** ^1^ Department of Cell and Developmental Biology, University of Colorado Anschutz Medical Campus, Aurora, CO, United States; ^2^ Department of Biochemistry and Structural Biology, University of Texas Health Science Center at San Antonio, San Antonio, TX, United States; ^3^ Department of Physics, University of Alberta, Edmonton, AB, Canada; ^4^ DIMEAS, Politecnico di Torino, Turin, Italy; ^5^ Department of Data Science and Engineering, The Silesian University of Technology, Gliwice, Poland

**Keywords:** microtubules, isotypes, evolution, therapeutic targets, structure-to-function relationship, tubulin

Tubulin, found in all eukaryotes, exists in many forms: *α*, *β*, *γ*, and others. *α* and *β* form a heterodimer, polymerizing into microtubules, often nucleated by *γ*. Not surprisingly, for a protein that has existed since eukaryotic cells appeared billions of years ago, *α*, *β*, and *γ* diverged into different forms---isotypes---with different amino acid sequences encoded by different genes. This Research Topic explores tubulin isotypes and addresses their evolutionary, functional and medical significance.

## Evolutionary significance of tubulin isotypes

For a better perspective we must dive into the origin of tubulin, which is itself a member of a superfamily, including the eubacterial FtsZ as well as other proteins found in many prokaryotes and even viruses. These proteins form filaments and bind to GTP and have similar primary and tertiary structures (reviewed in Ludueña, 2013).

An algorithm based on a model for the evolution of the genetic code, places FtsZ among the oldest 10 proteins, coming in third after ferredoxin and slightly older than proteins involved in nucleic acid metabolism (Davis, 1999; Davis, 2002). FtsZ participates in bacterial cell division and binds to membranes and actin filaments.

Fulton’s introductory essay explains the origin of the “multi-tubulin hypothesis,” the idea that there have to be distinct types of tubulin performing different functions. In *Naegleria*, a unicellular organism that goes from an amoeboid to a flagellated stage, the former contains microtubules that form the mitotic spindle, while the latter has them forming flagella. The Fulton laboratory showed, using ^35^S-methionine, that flagellar tubulin formed *de novo*, meaning that the mitotic spindle tubulins were not recycled into flagella, suggesting that there had to be more than one set of α- and β-tubulins. Gene sequencing revealed two α- and two β-tubulins in *Naegleria*, thus confirming the existence of isotypes. Fulton also suggests that an “engine” pushing the evolution of isotypes may be that duplication of tubulin genes would allow for more tubulin molecules to be produced as needed; later those genes could diverge into isotypes.

Bera and Gupta argue that microorganisms, being easily manipulated, are useful in studying isotypes. They present cases where isotypes have different functions, sometimes forming microtubules differing in dynamic behavior, temporal expression and subcellular localization.

Gasic reviews the eukaryotic realm to show relations among isotypes and to explore the regulation transcription of isotypes, including autoregulation. Gasic argues that isotypes exist to perform different functions and to be expressed in different cell types.

Lu and Zheng focus on *C. elegans*, which has the advantage of easy genetic manipulation and also of being transparent, allowing for microtubule behavior to be visualized in a living cell. Some isotypes are found in every tissue, some in just a few. Some form microtubules with 15 instead of the canonical 13 protofilaments. These authors describe the individual functions of each isotype and point out that tubulin’s numerous post-translational modifications add to the complexity and also may not be the same for different isotypes.

Based on these works, we can imagine that gene duplication caused the appearance of tubulin isotypes. When multicellular eukaryotes arose, it is conceivable that they had one isotype for mitosis and another for axonemes, both doing their jobs adequately. Gene duplication would have led to the appearance of more isotypes and gradual refinement as the jobs were distributed among the isotypes, followed by each one evolving to doing its job better. With more gene duplication, new isotypes appeared, each one more specialized, leading to further cell complexity and great variation of cell and tissue function, allowing multicellularity in eukaryotes.

## Functional significance of tubulin isotypes

A key prediction of the multi-tubulin hypothesis is that isotypes exhibit distinct functions, presumably imparted by different amino acid sequences and structures.

Montecinos et al. examine the drug-binding properties of chicken erythrocyte tubulin, which is enriched for the βVI isotype and encoded by the TUBB1 gene in humans. They show that tubulin containing βVI is distinct from mammalian brain tubulin in that it binds weakly to most drugs that target the colchicine site, but tighter to several benzimidazole compounds. This indicates a structural divergence of the colchicine site in βVI tubulin, and points to the potential of benzimidazoles, which are commonly used to target parasite tubulins, for further development of drugs that selectively target human isotypes.

Comparing the function and structure of individual isotypes has long been limited by the lack of isotypically pure sources of tubulin. The mini review by Ti recounts the long history of tubulin purification and reconstitution strategies, including recent breakthroughs that have enabled the expression and purification of recombinant, isotypically pure tubulin. These new approaches have opened avenues for comparing isotypes within or across species, and modeling tubulin variants linked to human disease.

The review by Sulimenko et al. extends the isotype comparison to another member of the tubulin family, the γ-tubulins. The human genome encodes two γ-tubulin genes that promote nucleation of αβ-tubulin assembly at centrosomes and other limited sites within cells. The authors describe recent advances in our understanding of the γ-tubulin structure and the mechanism of microtubule nucleation, and emerging functions of γ-tubulin in signaling and DNA repair within the nucleus.

## Medical significance of tubulin isotypes

In recent years, many links between tubulin isotypes and human disease have been established. These diseases are either associated with aberrant expression of isotypes or expression of isotypes containing missense mutations that alter function.

Phyo et al. introduce a tubulin code, which becomes rewritten to establish a proliferated, stable microtubule (MT) network that drives cardiac remodeling. They provide evidence of tunable tubulin autoregulation during pathological progression. This “tubulin code” is based on the permutations of tubulin isoforms and their post-translational modifications. A post-translationally-modified MT network is shown to underlie cellular growth in cardiac hypertrophy contributing to contractile dysfunction in heart failure. Using heart failure patient samples and murine models of cardiac remodeling the authors found that autoregulation occurs across tubulin isoforms in the heart concomitantly with rapid transcriptional and autoregulatory activation of specific tubulin isoforms and MT motors.

Buscaglia et al. show that reduced TUBA1A is sufficient to support neuronal migration and cortex development, which provides mechanistic insights into the MT function in support of neurodevelopment. The MT cytoskeleton drives neurite outgrowth, promotes neuronal growth cone responses, and facilitates intracellular cargo transport during neurodevelopment. Since TUBA1A constitutes the majority of α-tubulin in the developing brain, its mutations cause severe brain malformations associated with neurological tubulinopathies. The authors show that a TUBA1A loss-of-function mutation TUBA1A^N102D^ reduces its expression levels and prevents its incorporation into MT polymers. In mice, this leads to grossly normal brain formation except for a major impact on axon extension and impaired formation of forebrain commissures.

Luduena et al. discuss that contrary to normal cells, βII tubulin is present in the cytoplasm and nuclei of many tumor cells. Based on earlier work on nuclear βII, the authors suggest that the presence and location of βII in biopsies could be a useful prognostic indicator and that βII may be involved in cancer progression. They suggest that a signaling pathway in nearby cells causes βII to be synthesized and localized to their nuclei. Hence, the presence of βII in non-cancerous cells could indicate a nearby tumor. A complex which combines αβII with CRISPR-Cas9 could enter the nucleus of a cancer cell and, if guided to the appropriate gene, might destroy the cancer cell or make it less aggressive. These findings offer the possibility that βII localization can aid in cancer diagnosis, prognosis and therapy.

Cushion et al. provide an overview of the α/β-tubulin mutations’ involvement in tubulinopathies such as lissencephaly, microcephaly, polymicrogyria, motor neuron disease, and female infertility. The clinical features associated with these diseases have been attributed to the expression patterns of individual tubulin genes and their distinct functional roles. Additional impact of tubulin mutations on microtubule-associated proteins (MAPs) is emphasized by the authors. Depending on their effects on MTs, MAPs can be classified as polymer stabilizers (e.g., tau, MAP2, doublecortin), destabilizers (e.g., spastin, katanin), plus-end binding proteins (e.g., EB1-3, XMAP215, CLASPs) and motor proteins (e.g., dyneins, kinesins). This review analyzes mutation-specific disease mechanisms that influence MAP binding and their phenotypic consequences.

Duly et al. point out that tubulin expression is commonly dysregulated in cancer. This is significantly found to involve βIII-tubulin, which is encoded by the TUBB3 gene. Whereas in normal cells, TUBB3 expression is tightly restricted, and is found almost exclusively in neuronal and testicular tissues, its over-expression has been reported in various epithelial tumours. Importantly, this has been reported to have a strong correlation with drug resistance and aggressiveness of the neoplastic disease. The paper by Duly et al. is focused on the transcriptional and posttranscriptional regulation of TUBB3 in both normal and cancerous tissue. Better understanding of the mechanisms that control TUBB3 expression, especially in cancer is likely to spur the development of improved cancer therapies since tubulin is one of the key targets in a broad spectrum of cancers since various tubulin isotypes exhibit different affinities for microtubule-targeting agents as demonstrated earlier by Huzil et al. (2007).
